# Low back pain and physical activity – A 6.5 year follow-up among young adults in their transition from school to working life

**DOI:** 10.1186/s12889-015-2446-2

**Published:** 2015-11-12

**Authors:** Lars-Kristian Lunde, Markus Koch, Therese N. Hanvold, Morten Wærsted, Kaj B. Veiersted

**Affiliations:** National Institute of Occupational Health, Gydas vei 8, 0336 Oslo, Norway

**Keywords:** Low back pain, Physical activity, Young adults, Longitudinal

## Abstract

**Background:**

The association between leisure time physical activity and low back pain in young adults is unclear and is in the need of prospectively obtained evidence. This study examined the course of low back pain and the association between low back pain and leisure time physical activity in a cohort of young adults in their transition from school to working life.

**Methods:**

Both low back pain and leisure time physical activity was monitored over a 6.5 year period in 420 subjects starting out as students within hairdressing, electrical installation and media/design. The association between physical activity and low back pain was investigated through the follow-up period by using linear mixed models analysis.

**Results:**

Low back pain was significantly influenced by time and overall there was a decreasing trend of low back pain prevalence throughout the follow-up. Analysis showed a weak trend of decreasing low back pain with moderate/high physical activity levels, but this association was not significant.

**Conclusions:**

Low back pain decreased during follow-up with baseline as reference. Findings in our study did show non-significant trends of reduced low back pain with increased leisure time physical activity. Still, we could not support the theory of moderate/high levels of physical activity acting protective against low back pain in young adults entering working life. Our results, in combination with previous relevant research, cannot support a clear relationship between physical activity and low back pain for young adults. Thus, recommendations regarding effect of physical activity on reducing low back pain for this group are not clear.

**Electronic supplementary material:**

The online version of this article (doi:10.1186/s12889-015-2446-2) contains supplementary material, which is available to authorized users.

## Background

With over 100 million people reporting discomfort in muscles or joints within the European population, the prevalence of musculoskeletal disorders (MSD) is a severe problem [1]. As the fourth greatest cause of overall ill health and the leading cause of disability, this it is also a global concern [2]. In the working population, the presence of MSD may result in reduced work ability [3, 4] and an increased sickness absence [1, 5].

The Norwegian Directorate of Health recommends young adults (age 13–17) and adults (age 18+) a minimum of 30–60 min of daily leisure time physical activity (PA) to gain positive health benefits. This is a rather common advice and is also promoted as the 10 000 steps per day slogan [6].

Previous studies have not been able to produce consistent results on the association between PA and musculoskeletal pain [7, 8]. A cross-sectional study published in 2011 indicated that PA (increased frequency, duration and intensity) seemed to reduce level of general chronic pain in adults [9]. This was also later shown through a longitudinal study design, with five data collection points over a 12 month period. There seemed to be a relation between the two variables when close in time, indicating that subjects reported less pain at times when they reported more exercise [10]. The discussion will then be if the decrease in pain leads to more exercise or if increased exercise reduces the pain.

Studies have shown that pain occurring in children/adolescents often are persisting [11, 12] and it is suggested that pain formed in younger years may be related to pain in adulthood [13]. As part of The Young Finns study the researchers found low level of PA to be an independent risk factor for low back pain (LBP) in 24 to 39 year olds [14]. This relationship was also reported by others studying a similar age group [15] and concerning general pain in children [16]. Some of the few prospective studies carried out on school children have not been able to show that PA at baseline can predict LBP at follow-up [17, 18]. However, most studies on this topic are cross-sectional or have short follow-up periods and/or a limited frequency of time points during follow up. Thus, larger prospective studies focusing on leisure time PA and its association to LBP in young are lacking [7]. The prevalence of back pain in young increases with age [19] and entering working life may additionally introduce a set of risk factors for MSD [20–22], and therefore possibly increase the risk of developing pain [23]. A study following both level of PA and development of LBP in young adults during their transition from school into their first years of working life may give important information on causal relationships. Several authors suggest that young subjects should be focused on to prevent present and future LBP and that PA should be part of the solution [19, 24, 25]. However, the evidence on the role of PA in this setting is scarce and information based on longitudinal studies should be of importance when determining if PA may prevent LBP in young stepping into adult life [26, 27].

The overall aims of this study was to prospectively examine the prevalence and course of LBP in young adults transitioning from school to working life over a 6.5 year period. The specific objective was to investigate associations between PA level and LBP using multiple time point measurements.

## Methods

### Study design

This study was designed as a part of a larger prospective cohort study following students from 13 technical schools in the greater area in Oslo, Norway [28]. The students included studied either media/design, electrical installation or hairdressing. Data collection started in October 2002 and aimed to follow young adults in transition from school, via apprenticeship and into working life. The final data collection in this cohort was finished in February 2009. The baseline data collection was carried out at the different school sites and a sample of 420 out of the 496 invited volunteered to participate in the study. During the 6.5 year-follow-up each participant received a questionnaire approximately every 4th month, a total of 21 time-points (T0-T20). This study was approved by the Regional Committee for Medical and Health Research Ethics in Norway (S-02159 REK south-east). All subjects were given written information on the purpose and methods of the study and signed a written consent prior to participation. For subjects younger than 18 years at baseline parental consent was obtained in addition.

### Measures

#### Physical activity

The PA question monitored the frequency of leisure time activity the previous month. The question was: “How often do you perform activities that lead to increased heart rate and shortness of breath?“and had seven response options: 0 (never), 1 (less than once a month), 2 (once a month), 3 (once a week), 4 (2–3 times a week), 5 (4–6 times a week) and 6 (every day) [29]. These responses were dichotomized into responses of low (responses 0–3) and moderate/high (response 4–6) due to data distribution and to distinguish between high and low level of physical activity. Self-reported PA levels were collected at T0, T2, T4, T5, T7, T11, T14, T17 and T20.

#### Low back pain

Participants were asked for LBP the previous month at every time point (total of 21 repeated measures). The question was assisted by a drawing taken from the “Nordic questionnaire on musculoskeletal symptoms” [30] and measured pain intensity (no = 0, mild = 1, moderate = 2 and severe = 3) and pain duration (1–5 days = 1, 6–10 day = 2, 11–14 days = 3, and 15–28 days = 4) [31]. From answers on pain intensity and duration a pain index calculation was carried out by multiplying intensity (0–3) with duration, providing a pain score ranging from 0 to 12. This index has been used for this type of data previously [32] and has shown satisfying reliability [33]. The pain score from 0 to 12 for LBP was categorized into four levels of pain ranging from no pain (0), via mild (1) and moderate (2–3) to moderate/severe pain (≥4) which then was used in the statistical analysis.

#### Tobacco use

At baseline participants were asked for smoking habits and snuff use. They were registered as tobacco users if they smoked or used snuff daily or occasionally. The questions of these habits were repeated in questionnaires answered in total six times during the study (T0, T7, T11, T14, T17 and T20).

#### Ethnicity

Ethnicity of the individual was in this study based on parents’ country of birth. If both parents were born in a western country, ethnicity of the participant was stated as western. If one or both parents were born in a non-western country, the participant was considered as non-western.

#### Socioeconomic background

The socioeconomic background was based on the question “How wealthy do you consider your family?” with the response alternatives: very wealthy, wealthy, average wealthy, not particularly wealthy and not wealthy. Participants answering below average wealth were considered as low, while the remaining answers were categorized as medium/high socioeconomic background [29].

#### Body mass index

Body mass index (BMI) was calculated using self reported weight and height (kg/m^2^) and was obtained at time point T0, T7, T11, T17 and T20.

#### Physical work demands

The physical demands of work/school were assessed at baseline by the question: *How physically demanding do you find your work/school?* Response alternatives ranged from 0 (very, very easy) to 14 (very, very hard). Subjects responding in range 0–4, 5–9 and 10–14 were categorized in groups of low, moderate and high demands, respectively.

### Statistical methods

The questionnaire items used in this study were assessed with varying frequency over the 6.5 year follow-up period. The outcome variable *low back pain* was assessed at all 21 time points. The main exposure variable *physical activity* was assessed nine times in the same 6.5 year period. The time points including information on both main exposure and outcome (T0, T2, T4, T5, T7, T11, T14, T17, T20) were used for analysis (obs. = 2087). Data was checked for multicollinearity based on variance inflation factor and normality of the residuals was checked (in form of a normal quantile plot) from the mixed model analysis of one single imputed data set. To analyze differences between genders linear regression was implemented as an unadjusted, separate analysis. To examine the course of LBP both prevalence data and time variable coefficients from linear mixed models at each time point during follow-up was used. Adjusted linear mixed models with a random intercept and slope for each person were applied to study the association between PA and LBP. The random intercept allow for subject specific average pain levels, while the random slope allow for subject specific change in pain levels with time. Significance level was set at *p* < 0.05 and results are reported as coefficient with 95 % confidence interval. Statistical analysis was done using STATA 13.0 (StataCorp, College Station, TX, USA).

Handling of missing data was done by multiple imputation. Socioeconomic background, physical activity and ethnicity had small amount of missing in total (obs. = 44) and was therefore not imputed. Both BMI and tobacco status were not collected at time points T2, T4 and T5. In addition BMI were not collected at T14. At the time points collected, BMI had a moderate/large amount of missing (29 %) while tobacco use were almost complete (0.5 % missing). This resulted in BMI having 1287 (62 %) missing data points and tobacco status had 822 (39 %) missing data points for the 6.5 year period. Therefore, these variables were imputed by linear mixed models based on likelihood ratio tests using a criteria of *p* < 0.1. Due to a relatively high proportion of missing data a total of 40 imputed datasets were made [34]. The imputation of tobacco status and BMI enabled us to include the time points T2, T4, T5 and T14 in the analysis, and to retain a high sample size for the other time points. An estimated average dataset was calculated based on these imputed datasets and used for analysis. A detailed description of the multiple imputation procedure and its use in the mixed models analysis is provided as Additional file 1.

## Results

### Descriptive statistics

#### Subjects and response rates

At time of inclusion the 153 men and 267 women participating in the study had a mean age of 17.5 (SD ± 1.2). Participants’ individual characteristics at baseline are shown in Table 1. Questionnaire response rates varied from 100 % (*N* = 420) at T0 to 27 % (*N* = 113) at T18, with 44 % (*N* = 183) answering above half of all questionnaires and 7 % (*N* = 30) missing at all follow-ups. A total overview of collected observations for all variables at each time point is shown in Table 2.

**Table 1:** Baseline characteristics of the study population, stratified on gender

	Women (*n* = 267)	Men (*n* = 153)
	*Number and %*	*Number and %*
Tobacco status		
*No*	121 (45 %)	79 (52 %)
*Yes*	145 (54 %)	74 (48 %)
Education/profession		
*Media/design*	99 (37.1 %)	36 (23.5 %)
*Hairdresser/electrician*	168 (62.9 %)	117 (76.5 %)
Ethnicity		
*Western*	222 (84 %)	124 (82 %)
*Non-western*	43 (16 %)	28 (18 %)
Socioeconomic background		
*Low*	39 (15 %)	25(16 %)
*Moderate or high*	223 (85 %)	128 (84 %)
Low back pain		
*No*	111 (42 %)	84 (55 %)
*Low*	79 (30 %)	32 (21 %)
*Moderate*	30 (11 %)	13 (8 %)
*High*	45 (17 %)	24 (16 %)
Physical activity level		
*Low (≤1 pr wk)*	161 (60 %)	56 (37 %)
*Moderate/high (≥2 pr wk)*	106 (40 %)	97 (63 %)
Body mass index		
*Underweight (<18.5)*	27 (11 %)	18 (12 %)
*Normal (18.5–25)*	191 (74 %)	104 (68 %)
*Overweight (>25)*	39 (15 %)	31 (20 %)
	*Mean ± SD*	*Mean ± SD*
Physical work demands (1–14)	6.5 (±2.0)	5.5 (±2.5)

**Table 2:** Data collection procedure and numbers of observations

	2002–2003			2003–2004			2004–2005			2005–2006			2006–2007			2007–2008			2008–2009		
	T0^a^	T1	T2^a^	T3	T4^a^	T5^a^	T6	T7^a^	T8	T9	T10	T11^a^	T12	T13	T14^a^	T15	T16	T17^a^	T18	T19	T20^a^
Tobacco	419	______________________________						203	______________			198	_________		146	_________		138	_________		191
Education	420	________________________________________________________________________________________________________________________________________________																			
Ethnicity	417	________________________________________________________________________________________________________________________________________________																			
Socioeconomic	415	________________________________________________________________________________________________________________________________________________																			
Low back pain	418	266	290	259	278	262	238	206	212	197	181	196	166	142	145	134	141	138	113	143	191
Physical activity	420	___	298	___	285	263	___	206	______________			195	________		145	_________		138	________		192
BMI	410	_______________________________						140	______________			87	__________________________					78	________		132
Physical work demands	420	________________________________________________________________________________________________________________________________________________																			

^a^Time point included in analysis. Most participants with electrician/hairdresser education carried out their apprenticeship between T3 and T9. Between T10 and T20 most participants had entered working life

#### Course of low back pain and physical activity

**Fig. 1:**
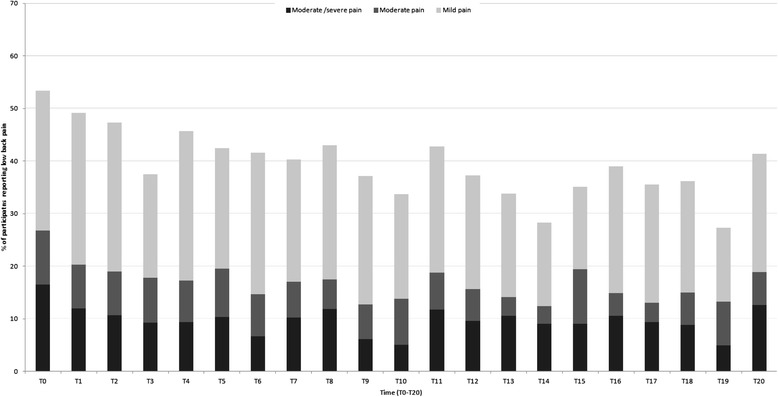
Repeated measures of low back pain during 6.5 year follow-up

At baseline (T0) 54 % reported to have any level of LBP the previous 4 weeks, with 26 % reporting mild pain, 10 % reporting moderate pain and 17 % reporting moderate/severe level of pain. Fifty-two percent reported to be physically active once a week or less at baseline, while 48 % reported to be physically active two times per week or more. The proportion of subjects with any level of LBP did show a decreasing trend from baseline through the follow-up, with range of prevalence of any level of LBP varying from 54 % at T0 (highest) to 27 % at T19 (lowest). Prevalence of moderate and moderate/severe pain was more stable during follow-up than prevalence of mild pain. The mean prevalence of any LBP for the follow-up period was 39 %. Level of PA showed an increasing trend during follow-up, with lowest level of reported moderate/high PA at T5 (44 %) and highest at T17 (59 %). Prevalence of subjects reporting different levels of LBP during follow-up are shown in Fig. 1.

#### Gender differences

**Fig. 2:**
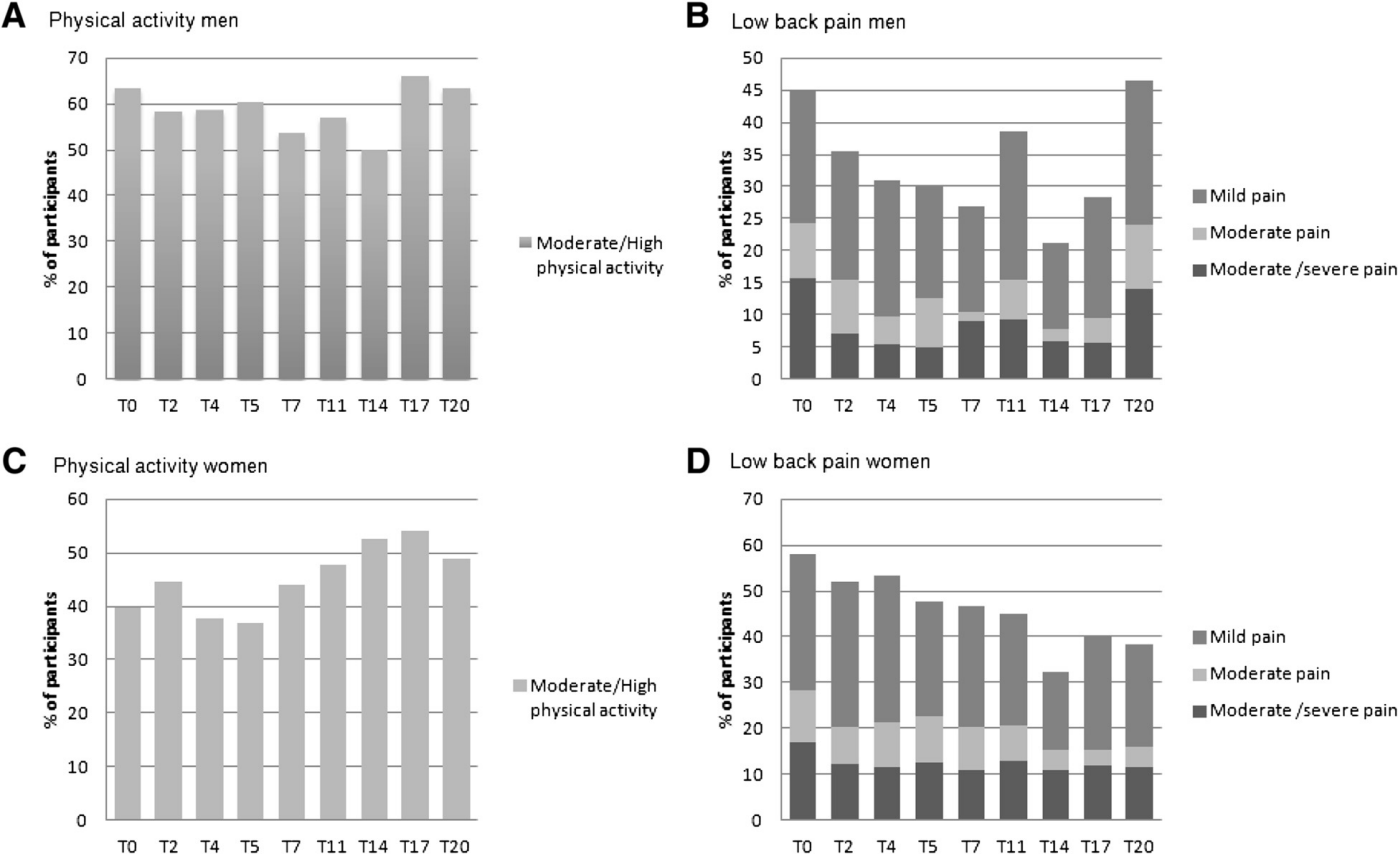
Low back pain prevalence and reported physical activity among male and female at measured time points. **a** shows prevalence of moderate/high level of physical activity ( ≥ 2 days per week) among men. **b** shows prevalence of low back pain in men (mild pain = 1, moderate pain = 2-3, moderate/severe = ≥ 4) **c** shows prevalence of moderate/high level of physical activity ( ≥ 2 days per week) among women. **d** shows prevalence of low back pain in women (mild pain = 1, moderate pain = 2-3, moderate/severe = ≥ 4)

Prevalence data showed that females reported higher proportion of LBP than males at all time points, except at T20, and that males reported higher levels of PA than females at all time points, excluding T14. Females had a mean prevalence of any LBP of 43 % (range 26–58 %), while men showed a mean prevalence of 31 % (range 18–47 %) of any LBP over the 6.5 year period. Linear regression analysis displayed no significant difference between males and females in reporting of LBP at baseline, T1 or T9-T20, but showed that females reported significantly higher levels of LBP than males at all time points from T2 to T8 (*p*-values < 0.05). Further, males had a significantly (*p*-values < 0.05) higher weekly level of PA compared to females at baseline. During follow-up men reported significantly higher levels of PA at all measured time points from T2 to T5 (*p*-values: <0.05). However, from T7 to T20 there were no significant differences between genders in reported PA level due to an increase in female PA. The courses of LBP and PA for both genders during this study are shown in Fig. 2.

### Associations to low back pain

#### All subjects

There was a significant (*p* < 0.01) association between time and LBP development. Compared to baseline there was a trend of reduction in reported pain at follow-up time points. The reduction in pain reporting with time was highest at T14 (coef. -0.48) and lowest at T3 (coef. -0.20). A weak protecting, but non-significant, association between moderate/high levels of PA on reported level of LBP was found in this study (−0.08, CI −0.16–0.01, *p* = 0.08). All results from the mixed model analysis are shown in Table 3.

**Table 3:** Adjusted mixed model analysis with low back pain as dependent variable

	All subjects			
Variables	Coefficient	*p*-value	95 % CI	
Gender				
*Man*	Ref			
*Woman*	0.22	0.10	−0.05	0.48
Age	0.06	0.08	−0.01	0.14
Tobacco status				
*No*	Ref			
*Yes*	0.09	0.17	−0.04	0.21
Education/profession				
*Electrician*	Ref			
*Hairdresser*	−0.07	0.64	−0.38	0.24
*Media/Design*	0.003	0.98	−0.26	0.27
Ethnicity				
*Non-western*	Ref			
*Western*	−0.09	0.50	−0.30	0.15
Socioeconomic background				
*Low*	Ref			
*Moderate or high*	0.01	0.92	−0.20	0.22
Physical activity level				
*Low*	Ref			
*Moderate/high*	−0.08	0.08	−0.17	0.01
BMI (kg/m^2^)	0.03	<0.01	0.01	0.05
Physical work demands				
*Low*	Ref			
*Moderate*	0.13	0.20	−0.07	0.33
*High*	0.23	0.27	−0.18	0.65
Time				
*T0*	Ref			
*T2*	−0.20	<0.01	−0.32	−0.08
*T6*	−0.24	<0.001	−0.37	−0.12
*T5*	−0.27	<0.001	−0.40	−0.14
*T7*	−0.29	<0.001	−0.43	−0.15
*T11*	−0.24	<0.01	−0.39	−0.09
*T14*	−0.48	<0.001	−0.65	−0.31
*T17*	−0.39	<0.001	−0.56	−0.21
*T20*	−0.24	<0.01	−0.40	−0.08

#### Gender stratification

For both men and women there was an overall trend of reduced LBP with time when using baseline as reference. For males there was a significant (*p* < 0.05) association with time with negative coefficients for all time points except T2, T11 and T20. This was also shown for females with a significant (*p* < 0.05) association with time reducing LBP reporting on all time points, with the highest coefficients towards the end of follow up, at T14, T17 and T20. As in unstratified analysis both genders showed non-significant trends of a reduction in LBP reporting with moderate/high levels of PA using low levels of PA as reference. For men this trend was very weak and not significant (−0.03, CI −0.17–0.12, *p* = 0.73). Women had a stronger association of moderate/high levels of PA on reported level of LBP category. Results indicated a reduction of pain reporting by −0.11 when going from low to moderate/high levels of PA. The values reflected for females was borderline non-significant (−0.11, CI −0.22–0.006, *p* = 0.06). All results from the stratified mixed model analysis are shown in Table 4.

**Table 4:** Adjusted mixed model analysis with low back pain as dependent variable, stratified on gender

	Men				Women			
Variables	Coef.	*p*-value	95 % CI		Coef.	*p*-value	95 % CI	
Tobacco status								
*No*	Ref				Ref			
*Yes*	0.07	0.43	−0.11	0.26	0.08	0.34	−0.08	0.24
Education/profession^a^								
*Electrician/Hairdresser*	Ref				Ref			
*Media/Design*	0.04	0.82	−0.29	0.36	0.07	0.48	−0.12	0.27
Age	0.05	0.57	−0.13	0.23	0.06	0.13	−0.02	0.14
Ethnicity								
*Non-western*	Ref				Ref			
*Western*	−0.20	0.33	−0.61	0.21	−0.04	0.787	−0.31	0.23
Socioeconomic background								
*Low*	Ref				Ref			
*Moderate/high*	0.19	0.31	−0.17	0.56	−0.08	0.54	−0.34	0.18
Physical activity level								
*Low*	Ref				Ref			
*Moderate/high*	−0.03	0.73	−0.17	0.12	−0.11	0.06	−0.21	0.01
BMI (kg/m^2^)	0.01	0.40	−0.02	0.05	0.03	<0.01	0.01	0.06
Work demands								
*Low*	Ref				Ref			
*Moderate*	0.02	0.89	−0.28	0.32	0.19	0.16	−0.07	0.46
*High*	0.26	0.48	−0.46	0.99	0.23	0.37	−0.28	0.74
Time								
*T0*	Ref				Ref			
*T2*	−0.19	0.06	−0.39	0.01	−0.20	0.01	−0.36	−0.04
*T4*	−0.32	<0.01	−0.52	−0.13	−0.20	0.02	−0.36	−0.03
*T5*	−0.32	<0.01	−0.53	−0.12	−0.24	<0.01	−0.40	−0.07
*T7*	−0.34	<0.01	−0.56	−0.12	−0.25	0.01	−0.43	−0.07
*T11*	−0.13	0.27	−0.37	0.10	−0.28	<0.01	−0.46	−0.09
*T14*	−0.36	<0.01	−0.62	−0.10	−0.53	<0.001	−0.75	−0.32
*T17*	−0.34	<0.05	−0.61	−0.07	−0.40	<0.01	−0.62	−0.17
*T20*	0.03	0.79	−0.22	0.29	−0.40	<0.001	−0.60	−0.19

^a^Due to small amount of female electricians (*n* = 5) and male hairdressers (*n* = 4) these professions are analyzed as one group in the stratified analyses

## Discussion

In this study we found a significant effect of time on LBP. Further, there was a weak protecting, but non-significant association between PA and LBP in this 6.5 year follow-up among young adults entering working life.

The overall decreasing trend in LBP from baseline to follow-up shown in this study has also been shown in other studies of LBP [35], and may be due to an initial attention being directed towards the pain area. Additionally, large population studies have shown that the prevalence of LBP is increasing from age 12 to around age 20, where it is seen a flattening of LBP reporting toward the age of 40 [19]. Even though the overall tendency was a decreasing level of LBP, males seemed to be more irregular in their reporting of LBP than females, thus the tendency was clearer amongst females. In our study we had a mean prevalence of LBP of 43 % amongst females and 31 % amongst males, which is somewhat higher than what is found in general for 17–24 year olds in the Norwegian working population [36]. The higher levels of reported LBP among young females compared to young males are shown in several studies [24, 37].

A recent review on the association between PA and LBP showed inconsistent results [7], being unable to conclude if PA could have negative or positive effects on development of LBP in adults. A study on adult cleaners which recorded PA and LBP weekly for a year found similar to our study a indicated weak protecting, but non-significant (*p* = 0.08) effect of PA [38]. In one of few longitudinal studies (3 year follow-up) on the relationship between PA and LBP in adolescents it was found at baseline and follow-up a lower frequency of PA and decreased strength amongst subjects with initial LBP [18]. On the contrary, a 4-year follow-up study on 10 to 19 year olds showed a significant relationship between increased PA and a more common history of LBP [39]. Others have also suggested that participation in sports may cause rather than prevent back pain in young, possibly in combination with growth. Still, the evidence for such relationships are lacking [27]. In adults it has been indicated that physical fitness rather than self-reported leisure time PA is more strongly related to LBP and that physical fitness measures could show clearer relationships [40]. In the study presented in this paper we did measure hand strength and shoulder endurance at baseline and found this to be significantly correlated to the self reported PA measure used. A study investigating the muscle strength on 5489 adolescent men at the age of 17–19 years could not relate low upper body muscular strength to self-reported musculoskeletal pain in adulthood 17 years later [41]. Thus, the authors were not able to confirm their hypothesis that low physical fitness was a risk factor for future musculoskeletal pain.

Generally the prevalence of any LBP had a decreasing trend during follow up, while the opposite was seen for PA levels. Gender stratification of data showed that females during the first years of follow-up (T0 – T5, 20 months) did have a significantly lower level of PA together with significantly higher level of LBP compared to males. An increase in PA in females starting at T7 (28 months) was seen together with a decrease in later reporting of any LBP, equalizing the differences between genders in reported PA seen from T0 to T5 and differences in LBP seen from T2-T8 (4 to 32 months). In Fig. 2c and d we see this increase in PA together with the decrease in LBP with time for females. Together with the borderline significance (*p* = 0.06) for PA on LBP in stratified analysis for females it is tempting to indicate a relationship. However, we do also see this reduction trend in LBP (despite higher level of variation in reporting) for males even though PA level are more stable in this group. Additionally it is important to not over interpret unadjusted descriptive data like the once seen in Fig. 2. Considering a potential latency in the effect of PA on LBP one could in future longitudinal studies evaluate the effect of PA at T0 on LBP at T1, effect of PA at T1 on LBP at T2 etc. (or similar strategies). In our study the time intervals between PA level collections differed, so a strategy using a discrete time variable with distinct time point effects was more appropriate.

A recent summary of previous reviews on interventions to reduce sedentary time for the young found that such interventions are generally successful, although effects seem to be small [42]. An intervention aiming to reduce LBP by home exercise and back health education in young did produce promising results with reduction in several LBP related outcomes after the intervention period of 12 weeks [43]. On the other hand, it is not from this study design possible to state if the reduction in pain was merely a result of healing with time. In our study we did see that the effect of time on LBP should be taken into consideration. With the design in our study we focused on information on PA and LPB taken pairwise from same time points over the whole follow-up period to obtain solid data concerning the association between these two variables. Thereby we have in this study a great foundation to address short-term effects over a long follow-up period. We approached the whole period by using the time variable, random effects of each participant and following the course of PA and LBP descriptively, but cannot determine if a certain level of physical activity may protect against LBP in the future. Others have shown positive short-term effects of different exercise programs to reduce LBP in adolescents, but have failed to determine any long-term effects [44, 45]. This may indicate that this relationship is in the need of continuous maintenance to bear fruit.

An obvious strength of this study is the longitudinal design with repeated measures of both exposure and outcome variable over a 6.5 year period. This gave us the opportunity to describe the course of low back pain over several years in young subjects in their transition from school into working life and further enabled us to look at the relationship between PA and LBP in this group over time. The investigated group of individuals in this study is of major importance, considering the lack of information on the potential effect of PA on LBP in this group and the importance of providing strategies to reduce musculoskeletal disorders in the population. Using multiple imputation for the BMI and tobacco variables made us able to replace missing values with plausible values providing less chance of biased estimates. The high number of imputations (*m* = 40) is relatively conservative with the goal of reducing power falloff [34].

The majority of both exposure and outcome data in this study is obtained through self-reports. This is a practical and efficient way to gather repeated measures over long periods of time. Still, it is well known that all self-reports may have limitations due to recalling and averaging values for previous weeks or month. Such issues may lead to both under and overestimating of the variables of interest [46]. In our study this may have affected both the main exposure variable PA and outcome variable LBP since both variables require the respondents to recall and average variable levels from the previous 4 weeks. Further, perceived level of strain considered as PA and perceived level of pain intensity may also be influenced by individual differences. However, initial misreporting should be of less concern when categorizing the variables in larger response groups prior to analysis, like done in this study. Even though such merging of response categories may lead to loss of information it is important to obtain a reasonable amount of participants in the response categories used. For the PA variable we argue that it is primarily of importance to differentiate between if low or high level of physical activity may have associations to low back pain, rather than between numbers of days being physically active. Variables directed towards concrete psychological aspects was not included in the adjusted linear mixed models in this study, something that could have affected the model output in an unknown manner due to its possible association to pain occurrence. We did however include variables concerning psychosocial dimensions that have previously shown to be associated with LBP [47]. We did have pronounced fall in response rate during follow-up from the initial high response rate at baseline, where 420 out of 496 invited responded. Some of the explanation to the relatively high loss to follow-up could be due to the initial questionnaire being provided in a school setting with time provided for this activity to be carried out. This may have lead to a higher proportion of participants initially, with the drawback of lower threshold for dropping out at a later stage due to lack of motivation. The high participation rate at baseline does however reduce possibility for a selection of subjects with e.g. high levels of LBP into the study. Possibility to generalize the findings in this study to other educational groups of young adults or to other nationalities is unknown, as there are differences between educational choices and origin of nationality when it comes to both health and lifestyle [29].

## Conclusions

We did in this study find a decreasing trend of low back pain during follow-up. Despite an indicated weak protective effect, analysis in our study did not support the theory of a protective effect of moderate/high levels of PA on LBP in young adults entering working life. With this study, we contribute to the investigation of the relationship between PA and LBP in young subjects during their transition from school to working life and during their first years of work. Our work, in combination with other relevant studies in this field cannot support a relationship between level of PA and development of LBP for this group. Thus, the recommendations to be given on leisure time PA in regards to LBP development in adolescents and young adults are not clear.

## Additional file

Additional file 1:
**Supplementary on multiple imputation.** (DOCX 22 kb)

## Abbreviations

BMI: Body mass index
LPB: Low back pain
MSD: Musculoskeletal disorders
PA: Physical activity
