# Supplementary material for: Low back pain and physical activity – A 6.5 year follow-up among young adults in their transition from school to working life
Source: BMC Public Health. 2015 Nov 12;15:1115. doi: 10.1186/s12889-015-2446-2 (PMC4643524; doi:10.1186/s12889-015-2446-2)
Supplement: Additional file 1: — Supplementary on multiple imputation. (DOCX 22 kb) [file 12889_2015_2446_MOESM1_ESM.docx]

**Supplementary file on multiple imputation strategy**

**For the article:**

***Low back pain and physical activity – A 6.5 year follow-up among young adults in their transition from school to working life***

**Description of Stata program**

The data were first reshaped from wide to long format retaining the nine follow-ups with data on both physical activity and low back pain. Records with missing data on low back pain, economy, ethnicity or physical activity were removed. Note that there is no need to impute the outcome variables (low back pain), as missing outcome values is best dealt with by the mixed model analysis itself.

Tobacco and BMI were each imputed based on mixed models. The same confounders as in the main analysis were considered: work demands, economy, education, ethnicity, gender, age and time point. In addition the outcome (low back pain) and exposure (physical activity) were considered for inclusion in the imputation models. Furthermore, we also allowed for a dependency between tobacco and BMI in their respective imputation models. Finally, considered possible interaction effects, as e.g. between age and gender. The final imputation models were chosen based on likelihood ratio tests, using a criteria of p<0.1 to ensure that the imputation model were not too restrictive.

For the tobacco variable, a logistic mixed model was first estimated for the observed, non imputed data. Work demands, physical activity and low back pain were included as fixed effects, while a random intercept for person was added. Then, missing tobacco status were imputed, based on estimated fixed effects, residual variance and predicted mean and variance for the random intercept.

A linear mixed model was estimated for observed BMI values. It included fixed effects for age, gender, tobacco status, education, ethnicity, low back pain and physical activity, while a random intercept and slope were added for each person. The estimates from this model were used to simulate missing BMI values in a similar way as for the tobacco data. As BMI depended upon the tobacco status, both the observed and imputed tobacco values were used in the imputation of BMI values.

Finally, the multiple imputed data were put in a format such that the Stata program recognizes it as imputed by the mi command in Stata. This enabled us to use the Stata tool “mi estimate” for the final mixed model analyses of the study data (MAMS data).

**Using the Stata imputation program in the mixed model analyses**

Our Stata program (setupMamsImputation, given below) creates a data set with multiple imputed values for BMI and tobacco status, taking the number of imputations as argument. Then we can use the “mi estimate” tool for the different mixed model analysis.

Example of stata command for the mixed model analyses, using multiple imputation:

**setupMamsImputation 40**

**mi estimate: mixed lowBackPain i.time i.physicalActivity i.gender c.age i.education**

**i.economy i.ethnicity i.tobacco BMI i.workDemands|| person:time, reml**

**Stata program for multiple imputation**

capture program drop setupMamsImputation

program define setupMamsImputation

args nimp

use "MAMS.dta", clear

quiet reshape long BMI@ physicalActivity@ lowBackPain@ tobacco@, **///**

i(person) j(time) **// Reshape to long format**

**// Keep follow-ups with (partial or complete) information**

**// on both exposure (physicalActivity) and outcome (lowBackPain)**

quiet keep if (time==0 | time==2 | time==4 | time==5 | time==7 | time==11 | ///

time==14 | time==18 | time==20)

quiet drop if (lowBackPain == .) **// drop records with missing outcome**

quiet replace economy = . if economy == 9

**// Remove observations with missing for either economy, ethnicity**

**// or physical activity(n=44).**

**// Only impute for BMI (n=1287) and tobacco (n=822):**

quiet drop if (economy == . | ethnicity == . | physicalActivity == .)

sort person time

**// Mixed model estimates for tobacco**

quiet meqrlogit tobacco i.physicalActivity i.lowBackPain ///

c.workDemands || person: **// including random slope (person:time) does not**

**// give reasonable BLUP's, hence only**

**// random intercept are included in the model**

**// Get BLUP's for the random effects for the tobacco logistic mixed model**

quiet predict ursblup*, reffects

**// Obtain standard deviations for the BLUP's**

quiet predict ursblup_se*, reses

matrix brs = e(b) **// Fixed effect estimates + SD of random intercept**

scalar sd_urs1 = exp(brs[1,11]) **// SD of random intercept**

**// Fix seed to ensure reproducible results**

set seed -77777

by person: gen occ = _n

**// Simulate missing values, store them temporary in BMIsim_i and**

**// tobacco_sim_i (i=1,2, ..., nimp)**

forvalues i = 1/`nimp' {

display _continue "."

**// ***** Part 1: Impute missing values for tobacco *******

**// Simulate random intercept (urs1)**

quiet gen urs1 = rnormal()*ursblup_se1 + ursblup1 if occ == 1

**// Fill in 5 missing values with values from estimated distribution for**

**// random effects (urs1), assuming zero mean**

quiet replace urs1 = rnormal(0,sd_urs1) if (urs1 == . & occ == 1)

**// Fill in random effect at all occasions for person**

quiet by person: replace urs1 = urs1[_n-1] if urs1 == .

**// Simulate the tobacco status BMI (using the imputed tobacco data)**

quiet gen logit_prob = brs[1,10] + brs[1,2]*(physicalActivity==1) + ///

brs[1,3]*(physicalActivity==2) + ///

brs[1,4]*(physicalActivity==3) + brs[1,6]*(lowBackPain==1) + ///

brs[1,7]*(lowBackPain==2)+ brs[1,8]*(lowBackPain==3) + ///

brs[1,9]*workDemands + urs1 //

quiet gen prob_rs = exp(logit_prob)/(1+exp(logit_prob))

quiet gen tobacco_sim_`i' = (uniform() < prob_rs)

**// Keep the observed values**

quiet replace tobacco_sim_`i' = tobacco if (tobacco < .)

**// ***** Part 2: Impute missing values for BMI *******

**// Mixed model estimates for BMI (using the imputed tobacco data)**

**// Assumed independance of random effects (cov(uns) not significant)**

quiet mixed BMI time i.gender age i.gender#c.age i.education i.ethnicity **///**

i.tobacco_sim_`i' i.physicalActivity i.lowBackPain || person:time, reml

**// Get BLUP's for the random effects for the BMI linear mixed model**

quiet predict ublup*, reffects **// BLUP's for the random effects**

quiet predict ublup_se*, reses **// SD for the BLUP's**

matrix b = e(b) **// fixed effect estimates + SD of resd**

scalar sd_e_ij = exp(b[1,27]) **// Get SD of residual variance**

scalar sd_u1 = exp(b[1,25]) **// Get SD of random slope**

scalar sd_u2 = exp(b[1,26]) **// Get SD of random intercept**

**// Simulate residuals, random intercept (u2) and random slope (u1)**

quiet gen eps = rnormal(0,sd_e_ij)

quiet gen u1 = rnormal()*ublup_se1 + ublup1 if occ == 1

quiet gen u2 = rnormal()*ublup_se2 + ublup2 if occ == 1

**// Fill in missing values (n=24) for random intercept (u2) and random slope**

**// (u1) with values from estimated distribution for random effects**

quiet replace u1 = rnormal(0,sd_u1) if (u1 == . & occ == 1)

quiet replace u2 = rnormal(0,sd_u2) if (u2 == . & occ == 1)

**// Fill in random effect at all occasions for person**

quiet by person: replace u1 = u1[_n-1] if u1 == .

quiet by person: replace u2 = u2[_n-1] if u2 == .

**// Impute BMI values based on estimated mixed model for BMI**

quiet gen BMIsim_`i' = b[1,24] + b[1,1]*time + b[1,3]*(gender==2) + ///

b[1,4]*age + b[1,6]*age*(gender==2) + b[1,8]*(education==2) + ///

b[1,9]*(education==3) + b[1,10]*(education==4) + ///

b[1,11]*(education==5) + b[1,13]*(ethnicity==1) + ///

b[1,15]*(tobacco==1) + b[1,17]*(physicalActivity==1) + ///

b[1,18]*(physicalActivity==2) + b[1,19]*(physicalActivity==3) + ///

b[1,21]*(lowBackPain==1) + b[1,22]*(lowBackPain==2) + ///

b[1,23]*(lowBackPain==3) + u1*time + u2 + eps

quiet replace BMIsim_`i' = BMI if BMI < . **// keep the observed values**

drop eps logit_prob prob_rs u1 u2 ublup1 ublup2 ublup_se1 ublup_se2 urs1

**}**

**// Impute dummy values for BMI and tobacco, to be replace by the simulated**

**// values above**

quiet mi set wide **// Creates _mi_miss**

quiet mi register imputed BMI tobacco

**// Dummy imputation, creating _1_BMI, _1_tobacco, _2_BMI, _2_tobacco,..**

quiet mi impute mvn BMI tobacco, add(`nimp')

**// Replace values imputed values by mi impute by those simulated by mixed models**

forvalues i = 1/`nimp' **{**

quiet replace _`i'_tobacco = tobacco_sim_`i'

quiet replace _`i'_BMI = BMIsim_`i'

drop tobacco_sim_`i' BMIsim_`i'

**}**

end
